# Do I feel elevated? Unraveling the emotional and value-based mechanisms behind humble leadership

**DOI:** 10.3389/fpsyg.2026.1847656

**Published:** 2026-09-01

**Authors:** Jieqi Shao

**Affiliations:** Heqin Honors School of Childhood Education, Ningbo Childhood Education College, Ningbo, China

**Keywords:** affective events theory, altruistic value, elevation, humble leadership, moral emotions, voice behavior

## Abstract

**Introduction:**

Humble leadership is increasingly recognized as a catalyst for organizational adaptability. Although prior research has examined several cognitive, relational, and affective mechanisms underlying humble leadership, less is known about whether humble leadership evokes specific self-transcendent moral emotions, such as elevation, that motivate followers to engage in risky but prosocial voice behavior. Departing from traditional exchange-based leadership perspectives, this study draws on Affective Events Theory to conceptualize humble leadership as a “moral event” that elicits elevation—a distinct, other-praising emotion triggered by witnessing moral excellence—and examines whether this emotional response motivates followers to engage in voice behavior, a risky and organization-oriented prosocial act. Furthermore, this study investigates whether follower altruistic value serves as a boundary condition that amplifies this process.

**Methods:**

A two-wave, dyadic design was employed with 313 leader-follower pairs from Chinese organizations—a cultural context where Confucian values and high power distance render humble leadership both counter-normative and morally salient. A moderated mediation model was tested using structural equation modeling in Mplus 8.0, with Monte Carlo simulation (20,000 resamples) to assess indirect effects.

**Results:**

Results demonstrate that humble leadership is positively related to follower voice behavior through the mediating role of elevation. Crucially, follower altruistic value strengthens this indirect effect, suggesting that the moral-emotional influence of humble leadership is more pronounced when it resonates with followers’ intrinsic values.

**Discussion:**

These findings contribute to the leadership and organizational behavior literature by identifying elevation as a theoretically meaningful moral-emotional pathway underlying follower voice, highlighting how moral emotions serve as an important emotional pathway for social influence in hierarchical organizations, and offering a culturally grounded account of how the Chinese organizational context shapes the emotional impact of humble leadership.

## 1 Introduction

Humble leadership is defined as “a manifested willingness to view oneself accurately, a displayed appreciation of others’ strengths and contributions, and teachability” (Owens and Hekman, 2012, p. 1,518). Rooted in moral and other-oriented values (Grenberg, 2005; Vera and Rodriguez-lopez, 2004), humble leadership has been shown to foster various forms of follower moral behavior, including voice (e.g., Ma et al., 2020; Naseer et al., 2020; Qin et al., 2021; Owens et al., 2019). Voice behavior, which entails the voluntary expression of constructive ideas, suggestions, and concerns intended to improve organizational functioning (Van Dyne and LePine, 1998; Morrison, 2011), is not only an extra-role behavior but also an act with significant ethical implications (Kish-Gephart et al., 2009). However, voice is distinct from many other positive workplace behaviors because it often requires employees to challenge existing practices and express potentially controversial opinions, creating personal risks such as negative evaluations or strained relationships with authorities (Morrison, 2011; Tangirala et al., 2013). Thus, engaging in voice requires employees to move beyond immediate self-protection and consider broader organizational interests, making voice a behavior that involves both personal risk and a concern for collective improvement (Tangirala et al., 2013). While accumulating research confirms that leadership style significantly influences voice (e.g., Wu and Zhou, 2024), a critical puzzle remains: how and why do morally grounded approaches, such as humble leadership, inspire followers to overcome these inherent risks and engage in morally courageous voice behavior? Responding to recent calls to examine leadership-voice dynamics through a “moral and ethical lens” (Dua et al., 2023), scholars are urged to identify the underlying motivational processes—beyond simple exchange relationships—that explain how ethical characteristics in leaders successfully ignite voice behavior.

In response to this theoretical gap, this study investigates a specific moral-emotional pathway through which humble leadership promotes followers’ voice behavior. By framing voice as a morally courageous act and humble leadership as an ethically driven influence, we aimed to deepen the understanding of how specific moral emotions explain why humble leaders can motivate followers to speak up despite the interpersonal risks associated with voice. Although previous research has substantially advanced our understanding of humble leadership by identifying cognitive and relational mechanisms through which humble leadership influences followers’ attitudes and behaviors, including psychological safety, empowerment, trust, and identification (Kelemen et al., 2022), these mechanisms primarily explain how humble leaders create supportive conditions that facilitate follower participation. Less is known about the specific moral emotions through which humble leadership motivates followers to engage in voice behavior, particularly when voice requires employees to move beyond immediate self-interest and consider broader organizational interests (Tangirala et al., 2013). Some studies have begun to examine affective processes associated with humble leadership and humility-related communication (e.g., Naseer et al., 2020; D’Errico, 2019), however, systematic examinations of how humble leadership evokes specific moral emotions and how these emotions motivate follower behaviors remain limited. For example, Naseer et al. (2020) empirically demonstrated that leader humility fosters followers’ positive moral emotions, specifically empathy and gratitude. However, these emotions primarily reflect followers’ appreciation of interpersonal relationships or received benefits, leaving unclear whether humble leadership can evoke self-transcendent moral emotions that inspire followers to act beyond self-interest. Notably, Shin et al. (2020) found that individuals from collectivist cultures may experience elevation in response to gratitude-related experiences, highlighting the importance of culturally embedded emotional processes. This observation points to a broader theoretical consideration: the cultural context in which humble leadership is enacted may shape how followers interpret and emotionally respond to leader humility. China provides a theoretically informative setting for examining this process because two cultural logics coexist. On the one hand, Confucian traditions value humility, moral self-cultivation, and concern for others as important ideals of exemplary leadership (Chan, 2015; Wang and Xiang, 2025). On the other hand, everyday organizational practice in high power-distance contexts often leads followers to expect leaders to maintain authority, preserve hierarchical distance, and demonstrate decisiveness (Farh et al., 2007; Hu et al., 2018). Thus, humble leadership may be valued as a moral ideal in Chinese culture while still being relatively unexpected in routine hierarchical leader–follower interactions. When leaders openly acknowledge limitations, seek subordinates’ input, and display teachability, such behaviors may therefore be interpreted as both relatively unexpected and morally admirable (Oc et al., 2015; Owens and Hekman, 2012). This coexistence of hierarchical expectations and Confucian virtue schemas provides a theoretical rationale for why humble leadership may be especially salient as a moral event in the Chinese organizational context. Importantly, because our study does not include a cross-cultural comparison, we present this cultural argument as a contextual interpretation rather than as empirical evidence that the effect is stronger in China than in other cultural contexts.

Building on these theoretical considerations, we identify elevation as the focal moral emotion in this study. Elevation, which arises when individuals witness acts of moral beauty or excellence (Algoe and Haidt, 2009), is particularly relevant to the humble leadership context for several reasons. Humble leaders, who openly acknowledge their own limitations and remain receptive to learning from others (Owens et al., 2013), may exemplify morally admirable behavior that resonates with followers’ moral sensibilities. These leaders’ humble, other-oriented, and self-effacing characteristics (Vera and Rodriguez-Lopez, 2004; Owens and Hekman, 2016) may help relieve followers’ psychological pressures and stimulate elevation. More importantly, elevation is particularly suited to explaining follower voice because its motivational orientation extends beyond personal appreciation of leaders and involves moral aspiration, self-transcendence, and concern for others’ welfare (Haidt, 2003; Algoe and Haidt, 2009). These characteristics closely align with the nature of voice behavior, which requires followers to move beyond immediate self-interest and contribute to organizational improvement despite potential interpersonal risks (Morrison, 2011; Tangirala et al., 2013). However, the extent to which humble leadership activates elevation, and whether this emotion mediates the link between humble leadership and follower voice behavior, has not been empirically examined. By focusing on elevation as a self-transcendent moral-emotional mechanism, this study advances theoretical understanding of how humble leadership influences follower voice through a process of moral inspiration rather than merely through cognitive evaluations or relational exchanges. Thus, our study extends humble leadership theory by identifying elevation as a complementary and relatively proximal moral-emotional pathway that operates alongside, rather than replaces, established cognitive, relational, and exchange-based explanations of follower voice.

Recent theoretical perspectives suggest that individuals are naturally motivated to seek harmony in their psychological relationships, a phenomenon described by Heider’s (1958) balance theory. According to this theory, people prefer balanced states in which their evaluations of others and their own internal beliefs are aligned, thereby reducing cognitive tension and fostering a sense of psychological balance. In organizational contexts, such balanced states may emerge when followers perceive leaders as holding values or displaying behaviors that reflect their own moral orientations. Specifically, when followers with strong altruistic values interact with humble leaders who embody other-oriented behaviors, such as appreciating others’ contributions and emphasizing collective interests over personal interests, they are more likely to view these leaders as morally exemplary. This perception fosters a psychologically balanced state that enhances followers’ receptivity to leaders’ influences. Under such conditions, followers are more sensitive to the affective signals embedded in humble leadership behaviors and thus are more likely to experience elevation, a positive other-directed moral emotion evoked by witnessing moral excellence (Algoe and Haidt, 2009). As a result, the emotional impact of humble leadership may be amplified among those who prioritize altruism, ultimately increasing the likelihood of elevation and prosocial behaviors, such as voice. Although recent studies have emphasized the importance of identifying boundary conditions that shape the effects of humble leadership (Kelemen et al., 2022), empirical work on follower-based moderators, especially those grounded in personal values like altruism, remains scarce. Followers often perceive humble leaders as being genuinely committed to working collaboratively for the collective good (Oc et al., 2015). Such behavior reflects a form of other-oriented leadership grounded in altruism (Argandona, 2015; Qin et al., 2021). Altruistic value, defined as a deep concern for others and society’s well-being, represents a key dimension of self-transcendence in value theory (Schwartz et al., 2001) and is known to influence individual motivation and behavior (Rahman and Reynolds, 2016). It guides individuals to prioritize others’ welfare (Rahman and Reynolds, 2016) and shapes how they respond to leadership cues. However, a critical question remains unanswered: are followers who endorse altruistic values more emotionally responsive to humble leaders and thus more likely to engage in voice behavior? This study addressed this gap by examining whether follower altruistic value intensifies the emotional and behavioral effects of humble leadership through the mediating role of elevation.

**Figure 1:**
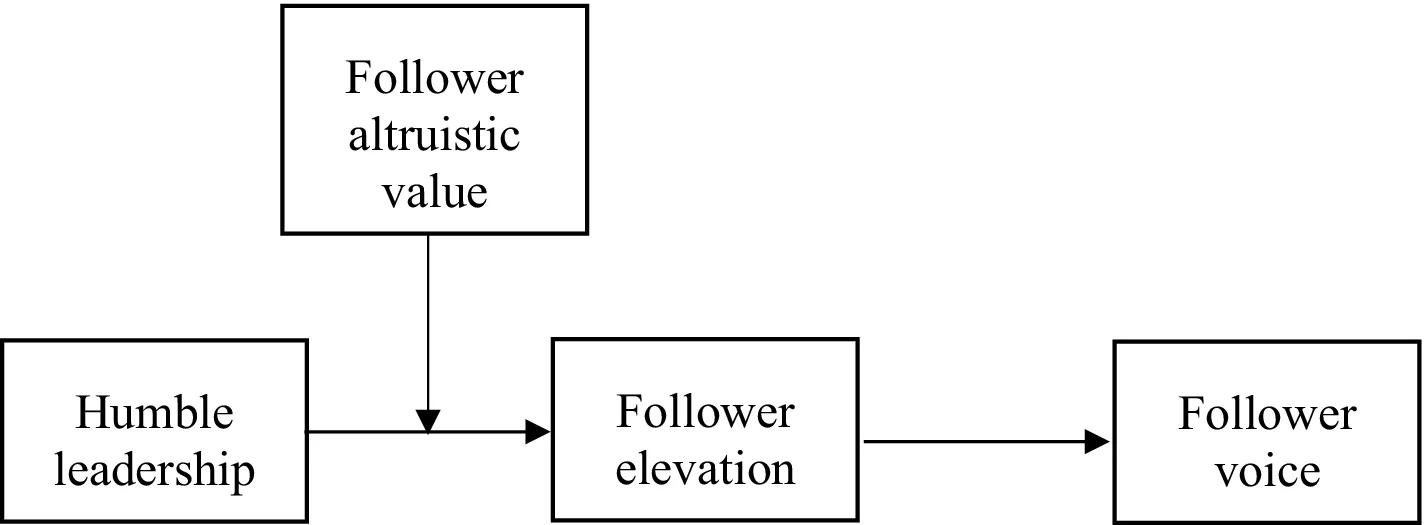
Research framework.

We proposed and tested a moderated mediation model that integrates a moral emotional pathway (elevation) and a key follower characteristic (altruistic value) into the leadership-voice behavior framework to deepen our understanding of how and when humble leadership fosters follower voice. First, our study advances humble leadership research by identifying a self-transcendent moral-emotional pathway through which humble leadership is associated with follower voice behavior. Prior studies have primarily explained the effects of humble leadership through cognitive and relational mechanisms, such as psychological safety, empowerment, trust, and identification (Kelemen et al., 2022). These perspectives have substantially advanced our understanding of how humble leaders create supportive environments that reduce barriers to follower participation. However, they provide limited insight into why followers may voluntarily engage in voice when such behavior requires challenging existing practices and involves interpersonal risks. By introducing elevation as a self-transcendent, other-praising moral emotion elicited by witnessing moral excellence (Haidt, 2003; Algoe and Haidt, 2009), we identify a theoretically grounded motivational pathway through which followers may become inspired to contribute beyond self-interest. Thus, our study extends humble leadership theory from a primarily cognitive and relational perspective toward a moral-emotional perspective. Second, we contribute to culturally grounded leadership theorizing by providing a contextualized examination of the humble leadership–elevation–voice pathway in the Chinese organizational context. Rather than treating culture merely as a sample characteristic, we theorize how specific cultural dimensions shape the proposed mechanisms. In high power-distance societies such as China, leaders are conventionally expected to project authority and decisiveness (Farh et al., 2007; Hu et al., 2018), making humble behaviors a conspicuous departure from the norm that heightens their moral salience. Simultaneously, the Confucian emphasis on *qianxu* (humility) as a core virtue (Kim, 2023; Chan, 2015) provides a cultural schema through which followers can readily interpret leaders’ humility as genuine moral excellence rather than weakness—a distinction that may not hold in individualistic cultures where humility can be conflated with a lack of confidence (Tangney, 2000; Kim, 2023), theoretically strengthening the affective pathway we propose. By explicating these culturally embedded processes, we move beyond the simple observation that research has undersampled non-Western contexts and instead offer a theoretical rationale for why the Chinese organizational context provides a theoretically informative setting for understanding how humble leadership translates into follower moral emotions and voice behavior, thereby addressing calls to integrate cultural values into leadership theorizing (e.g., Luo et al., 2022). Third, by identifying follower altruistic value as a boundary condition, we clarify the “moral resonance” required for leadership effectiveness. We show that followers with high altruistic values are more responsive to humble leaders, exhibiting stronger elevation and voice. This directly addresses Morrison’s (2014) call for contingency factors, demonstrating that the power of humble leadership lies not just in the leader’s behavior, but in its alignment with the follower’s moral compass. Figure 1 illustrates our proposed research framework.

## 2 Literature review

### 2.1 Humble leadership and follower voice

Within organizations, humble leaders openly recognize their shortcomings, value employee input, and express genuine interest in others’ ideas and growth (Owens and Hekman, 2012; Owens et al., 2013). These behaviors reflect the three core dimensions of humble leadership: accurate self-awareness, the appreciation of others, and teachability. Importantly, such behaviors are not merely interpersonal tactics; they embody an ethical orientation rooted in self-transcendence and moral regard (Grenberg, 2005). Humility fosters a relational dynamic grounded in authenticity and respect, laying the moral groundwork for prosocial and constructive employee behaviors. Thus, scholars have increasingly examined the ethical consequences of humble leadership (e.g., Owens et al., 2019; Qin et al., 2021). Humble leadership has been found to foster follower ethical behavior by enhancing moral self-efficacy (Owens et al., 2019) and promoting workplace spirituality (Naseer et al., 2020). Moreover, humility is viewed as a foundational virtue that reinforces managerial moral standards and collective development (Argandona, 2015). This moral dimension of humility may carry particular weight in cultural contexts where virtue-based leadership is deeply valued. In Confucian societies, for example, the ideal leader is expected to cultivate personal virtue as the foundation for governing others (Ou et al., 2014), rendering humble behaviors not merely admirable but morally expected of exemplary leaders—and thus especially powerful in eliciting follower prosocial responses.

Voice behavior, which refers to the discretionary communication of constructive suggestions to improve organizational functioning (Van Dyne and LePine, 1998), often involves personal risk (Morrison, 2011) and thus requires psychological safety and moral encouragement. Humble leaders provide this supportive context by creating an environment in which followers feel recognized, valued, and secure. They demonstrate openness to input and feedback even when it is critical, which signals followers that it is safe to express concerns and offer ideas (Owens et al., 2013; Bharanitharan et al., 2019). Humble leaders embrace uncertainty to foster an environment that bolsters followers’ confidence and perception of safety, motivating them to contribute novel ideas. Furthermore, humble leaders often highlight and value followers’ strengths and contributions, which enhances followers’ self-worth and competence perceptions (Owens et al., 2013; Rego and Simpson, 2018). When humble leaders are willing to identify and appreciate the unique expertise of their followers and actively seek their feedback, this role reversal boosts followers’ sense of self-efficacy (Owens and Hekman, 2012), reinforcing that their voice matters, which can lead to positive change. In sum, humble leadership fosters psychological safety and intrinsic motivation that empower followers to exhibit voice behavior for the greater good.

*H1*: Humble leadership is positively related to follower voice.

### 2.2 Humble leadership, follower elevation, and follower voice

According to affective events theory (AET; Weiss and Cropanzano, 1996), certain work events trigger followers’ fluctuating affective reactions; then, followers’ affect-driven behaviors directly result from these affective reactions. Some scholars have proposed that leaders are “proximal causes” of changes in followers’ emotional states (Weiss and Cropanzano, 1996, p. 31). A set of behaviors exhibited by humble leaders, such as accurate self-assessment, the appreciation of others’ strengths, and a willingness to learn, helps cultivate a positive work environment characterized by interpersonal fairness and an orientation toward others. Notably, leaders’ interpersonal fairness has been shown to strongly elicit elevation among followers (Vianello et al., 2010).

First, humble leadership provides the specific “moral stimuli” necessary to elicit elevation. Elevation is a specific moral emotion triggered by witnessing acts of moral beauty or excellence, characterized by a sensation of warmth and a desire to become a better person (Algoe and Haidt, 2009; Greenbaum et al., 2020; Vianello et al., 2010). Humble leaders exhibit moral virtues by objectively acknowledging their limitations, appreciating followers’ strengths, and prioritizing others’ development over their own status (Owens et al., 2013; Owens and Hekman, 2016). Such behaviors are perceived as self-effacing and other-oriented (Vera and Rodriguez-lopez, 2004), distinguishing them from standard management practices. Unlike general positive emotions (e.g., joy) or other-praising emotions like gratitude (triggered by direct benefits) and admiration (triggered by non-moral competence) (McCullough et al., 2001; Algoe and Haidt, 2009), elevation is specifically evoked by moral excellence. Importantly, elevation is conceptually different from other positive emotions that have been examined in leadership and organizational research. Although gratitude, admiration, inspiration, and general positive affect may all generate favorable behavioral outcomes, they represent different emotional experiences with distinct eliciting conditions and motivational orientations. Gratitude typically arises when individuals recognize that they have received valuable benefits or kindness from others and primarily motivates reciprocal or relationship-maintaining responses toward benefactors (McCullough et al., 2001; Algoe and Haidt, 2009). Admiration is generally elicited by witnessing exceptional competence, achievement, or excellence and motivates individuals to improve their own abilities or performance (Algoe and Haidt, 2009). Inspiration is typically conceptualized as a positive motivational state triggered by encountering outstanding accomplishments, abilities, ideas, or possibilities and primarily encourages individuals to pursue or enact a higher possibility (Thrash and Elliot, 2003; Algoe and Haidt, 2009). In contrast, elevation is specifically elicited by witnessing moral beauty or excellence and motivates individuals to become less self-focused, pursue moral self-improvement, and engage in prosocial actions that benefit others (Haidt, 2003; Algoe and Haidt, 2009). Therefore, elevation provides a complementary explanation for why followers may engage in voice behavior as a morally meaningful contribution to organizational improvement rather than merely as a response to personal benefits, admiration for leader competence, inspiration toward personal goals, or general positive feelings.

Beyond these affective and motivational distinctions, our focus on elevation is not intended to replace established cognitive, relational, or obligation-based explanations of the humble leadership–voice relationship. Rather, its theoretical value lies in explaining the moral motivation that emerges when followers must transcend immediate self-interest and contribute to collective outcomes. Consistent with affective events theory (AET; Weiss and Cropanzano, 1996), we conceptualize elevation as a complementary and relatively proximal moral-emotional pathway that operates alongside these mechanisms. Psychological safety and trust primarily explain how humble leaders reduce interpersonal risk and relational uncertainty, thereby making voice behavior feel safer and more feasible (Edmondson and Bransby, 2023; Gao et al., 2011; Kelemen et al., 2022). Felt obligation explains voice in terms of reciprocity, indebtedness, or a perceived responsibility to respond to favorable leader treatment (Duan et al., 2022). Elevation, by contrast, captures followers’ moral appraisal of leaders’ virtuous behaviors and the other-praising emotional response elicited by witnessing moral excellence (Haidt, 2003; Algoe and Haidt, 2009; Hericher et al., 2023). Elevation is also diifferent from moral awe. Although awe and elevation are both self-transcendent emotions that may motivate prosociality (Pizarro et al., 2021), awe centers on perceived vastness and the need for accommodation (Keltner and Haidt, 2003), whereas elevation centers on moral beauty, warmth, uplift, moral self-improvement, and the desire to emulate moral excellence through prosocial action (Haidt, 2003; Algoe and Haidt, 2009). Thus, elevation helps explain why followers may become morally inspired to engage in voice behavior as a constructive contribution to organizational improvement, rather than merely because they feel safe, trusting, obligated, or generally positive. Table 1 shows conceptual distinctions between elevation and related constructs.

**Table 1:** Conceptual distinctions between elevation and related constructs.

Construct	Definition / eliciting condition	Primary motivational orientation	Why it differs from elevation in explaining follower voice
Gratitude	A moral emotion that arises when individuals perceive themselves as beneficiaries of another person’s intentionally provided benefit or kindness (McCullough et al., 2001; Algoe and Haidt, 2009).	Reciprocity, acknowledgement, and relationship maintenance toward benefactors (McCullough et al., 2001; Algoe and Haidt, 2009).	Gratitude explains followers’ desire to reciprocate leaders’ support or kindness, but it less directly explains why followers engage in voice as moral emulation of leader virtue beyond interpersonal exchange.
Admiration	An emotion elicited by observing non-moral excellence, such as exceptional competence, achievement, or skill in others (Algoe and Haidt, 2009).	Emulation, learning, self-improvement, and enhancement of personal capability (Algoe and Haidt, 2009).	Admiration motivates followers to emulate leaders’ abilities or achievements, but it is primarily competence-oriented rather than focused on moral aspiration or prosocial organizational contribution.
Inspiration	A positive motivational state evoked by perceived higher possibilities, often encountered through outstanding ideas, accomplishments, or abilities (Thrash and Elliot, 2003; Algoe and Haidt, 2009).	Goal pursuit, aspiration, and enactment of higher possibilities (Thrash and Elliot, 2003; Algoe and Haidt, 2009).	Inspiration may encourage followers to pursue challenging goals, but it does not necessarily involve moral appraisal of leader virtue or other-oriented prosocial motivation.
Positive affect	A broad positive affective state involving pleasant feelings and general positivity; positive emotions can broaden individuals’ momentary thought-action repertoires (Fredrickson, 2001).	Broadened thought-action repertoires and resource building (Fredrickson, 2001).	Positive affect is too general to explain why followers engage in morally meaningful and potentially risky voice behavior, because it does not specify a moral elicitor or moral motivational orientation.
Moral awe / awe	A self-transcendent emotion elicited by perceived vastness and the need for accommodation; moral awe may arise when perceived vastness is associated with extraordinary virtue or moral greatness (Keltner and Haidt, 2003; Pizarro et al., 2021).	Reverence, small-self, accommodation, expanded perspective, and connection to something larger than the self (Keltner and Haidt, 2003; Pizarro et al., 2021).	Moral awe may involve reverence toward moral greatness, whereas elevation more specifically involves warmth, uplift, moral self-improvement, and the desire to emulate moral excellence through prosocial action.
Psychological safety	A cognitive-relational belief that speaking up is interpersonally safe and unlikely to result in punishment, embarrassment, or rejection (Edmondson, 1999; Edmondson and Bransby, 2023).	Risk reduction, openness, and willingness to express ideas or concerns (Edmondson, 1999; Edmondson and Bransby, 2023).	Psychological safety explains why voice feels safer or less threatening, whereas elevation explains why followers may feel morally inspired to voice as a constructive contribution to organizational improvement.
Trust	A relational belief that the leader is benevolent, reliable, competent, or has integrity, which increases followers’ willingness to be vulnerable (Mayer et al., 1995; Gao et al., 2011).	Reduced relational uncertainty, confidence in the leader–follower relationship, and willingness to accept vulnerability (Mayer et al., 1995; Gao et al., 2011).	Trust explains confidence in the leader or leader–follower relationship, whereas elevation explains moral inspiration arising from perceived leader virtue.
Felt obligation	A perceived duty or indebtedness to reciprocate favorable leader treatment or support (Eisenberger et al., 2001; Duan et al., 2022).	Reciprocity, repayment, and fulfillment of perceived duty or responsibility toward the leader or organization (Eisenberger et al., 2001; Duan et al., 2022).	Felt obligation explains voice as repayment or duty toward the leader, whereas elevation explains voice as moral emulation and prosocial contribution beyond indebtedness.
Elevation	A self-transcendent, other-praising moral emotion elicited by witnessing moral beauty or exceptional moral virtue in others (Haidt, 2003; Algoe and Haidt, 2009; Hericher et al., 2023).	Moral aspiration, self-transcendence, moral self-improvement, and prosocial action directed toward others (Haidt, 2003; Algoe and Haidt, 2009).	Elevation provides a distinct explanation for why followers internalize leaders’ moral exemplarity and become motivated to engage in constructive voice behavior beyond self-interest considerations.

When followers witness leaders openly admitting mistakes, acknowledging limitations, appreciating followers’ strengths and contributions, or displaying teachability (Owens et al., 2013; Owens and Hekman, 2012), they may perceive these actions as demonstrations of moral beauty that can elicit elevation. The potency of these moral stimuli may also be shaped by cultural context. In the Chinese organizational context, humility can be understood at two levels. At the level of cultural ideals, Confucian traditions emphasize moral self-cultivation, self-restraint, and the ideal of the *junzi* as a morally exemplary person who shows concern for others (Chan, 2015; Wang and Xiang, 2025). At the level of everyday hierarchical organizational practice, however, leaders are often expected to assert authority, maintain social distance, and project decisiveness (Farh et al., 2007; Hu et al., 2018). Thus, humble leadership is not inconsistent with Chinese cultural ideals; rather, it may be morally valued as an ideal while still being relatively unexpected in routine leader–follower interactions within hierarchical organizations. Against this backdrop, humble behaviors—such as openly acknowledging limitations and actively seeking subordinates’ input—may capture followers’ attention and be appraised as morally meaningful acts (Oc et al., 2015; Owens and Hekman, 2012). We therefore theorize that the coexistence of Confucian virtue schemas and high power-distance organizational norms may make humble leadership particularly salient as an affective event in Chinese organizations.

Second, elevation may help explain why followers become more willing to engage in voice behavior. According to AET, affective reactions lead to specific immediate behavioral responses (Weiss and Cropanzano, 1996). Elevation is theorized to involve a specific moral motivational process. Rather than merely generating positive feelings, elevation involves a self-transcendent motivational process that shifts individuals’ attention away from self-focused concerns toward moral ideals and collective welfare (Algoe and Haidt, 2009; Siegel et al., 2014). By fostering a more positive view of humanity and a greater sense of connection with others (Freeman et al., 2009; Oliver et al., 2015), elevation reduces individuals’ emphasis on personal benefits and encourages them to act in ways that support others’ welfare (Haidt, 2003). In organizational contexts, such a shift may lead followers to focus less on personal gains or relational costs and more on contributing to collective improvement. Such a motivational shift helps explain why followers may voluntarily engage in voice even when the behavior provides limited immediate personal returns. This self-transcendent motivational orientation is particularly relevant to voice behavior because voice requires employees to balance organizational interests against potential personal costs and to move beyond immediate self-protection when challenging the status quo (Morrison, 2011; Tangirala et al., 2013). In this sense, the motivational orientation of elevation closely corresponds to the behavioral demands of voice: both involve moving beyond immediate personal interests and directing attention toward broader collective outcomes. Empirical evidence further suggests that elevation can promote morally relevant prosocial behaviors, such as charitable donations to disadvantaged outgroups and employee behaviors that support CSR directed at secondary stakeholders (Freeman et al., 2009; Hericher et al., 2023). Thus, unlike gratitude, admiration, or general positive affect, which may facilitate favorable responses through reciprocity, competence recognition, or general positivity (Algoe and Haidt, 2009; Fredrickson, 2001), elevation may motivate followers to engage in voice because such behavior represents an opportunity to contribute to broader organizational welfare and enact the moral values inspired by humble leaders. In the present context, this suggests that elevated followers may be more willing to engage in voice behavior as a constructive contribution to organizational improvement, even though voice can involve interpersonal risks (Morrison, 2011). Voice behavior, defined as challenging the status quo to improve the organization, incurs personal risk but is fundamentally a prosocial and morally courageous act (Organ et al., 2006; Kish-Gephart et al., 2009; Morrison, 2011). Elevated followers, inspired by their leaders’ humility, are more likely to transcend personal risk calculations and engage in voice to promote organizational well-being (Tangirala and Ramanujam, 2008), thereby mirroring the moral excellence they have witnessed.

Based on this logic, we posit that humble leadership is indirectly associated with follower voice through a specific self-transcendent moral-emotional pathway: elevation.

*H2*: Follower elevation mediates the relationship between humble leadership and follower voice.

### 2.3 The moderating role of follower altruistic value

Values guide individuals’ attitudes and behaviors in organizations and influence the relationship between leaders and followers (Brown and Treviño, 2009). Some researchers have used follower values as boundary conditions when exploring the impact of leadership on followers’ moral reactions (e.g., Gebert et al., 2016; Islam et al., 2021). Altruistic value is a value structure that can significantly influence individual behavior. It is mainly manifested as concerns for social and others’ well-being (Rahman and Reynolds, 2016). In the workplace, followers who endorse high levels of altruistic value are more inclined to selflessly help others and prioritize collective interests over personal gains (Lee et al., 2014). Humble leadership is similarly characterized by an other-oriented and altruistic focus, as humble leaders appreciate followers’ contributions, demonstrate teachability, and emphasize the value of others above themselves (Owens et al., 2013; Oc et al., 2015). When followers with high levels of altruistic value interact with humble leaders who embody altruistic attributes, this perceived similarity can create a sense of psychological balance. The balance theory (Heider, 1958) holds that individuals are motivated to maintain balanced states in which their feelings and perceptions about others are consistent and free from tension. Experiencing this balance reinforces the perception of the leader’s moral excellence and increases followers’ receptiveness to positive affective cues, making them more likely to experience elevation in response. As a result, followers’ altruistic value can strengthen the emotional impact of humble leadership, enhancing the likelihood of experiencing elevation. Thus, we propose the following hypothesis:

*H3*: Follower altruistic value moderates the relationship between humble leadership and follower elevation; so, the relationship is stronger when follower altruistic value is higher.

### 2.4 An integrated moderated-mediation model

Based on the AET (Weiss and Cropanzano, 1996), humble leadership behaviors create positive and morally excellent work events that evoke followers’ other-directed moral emotion (i.e., elevation). As established in previous sections, this elevation motivates followers to engage in prosocial and extra-role behaviors, such as voice, which contributes to organizational well-being despite its inherent risks. Followers’ altruistic value strengthens this process by enhancing their sensitivity to the moral excellence of humble leadership, making them more likely to experience elevation in response. Consequently, followers with high levels of altruistic value not only experience stronger emotional reactions but are also more likely to translate those feelings into voice behavior. This suggests that altruistic value moderates the indirect relationship between humble leadership and follower voice through elevation; so, the mediated effect becomes stronger when follower altruistic value is higher. Thus, we propose the following hypothesis:

*H4*: Follower altruistic value moderates the relationship between humble leadership and follower voice through follower elevation; so, the indirect effect is stronger when follower altruistic value is higher.

## 3 Methods

### 3.1 Samples and procedures

The study collected data from 25 organizations located in Zhejiang Province, China, using leader–follower dyads. The participating organizations represented multiple industries, including energy and petroleum services, automobile sales and maintenance, banking and securities, professional services, vehicle inspection services and other local service organizations. Participants were recruited from different organizational levels and functional departments, including administrative, financial, operational, and professional departments. Before data collection, one author contacted several personal contacts working in different organizations and asked whether their organizations would be willing to participate in this study. These contacts subsequently communicated with their organizations’ human resource managers regarding participation. After obtaining organizational consent, participating organizations facilitated access to leaders and followers who met the study requirements. Specifically, followers were included when they had an identifiable direct leader who was familiar with their workplace behaviors, whereas leaders were included when they directly supervised participating followers and were able to evaluate their followers’ voice behavior. This convenience sampling approach through organizational contacts has been widely used in prior organizational research (Bavik et al., 2017).

To reduce common method bias (Podsakoff et al., 2012), we employed a two-wave, time-lagged, and multisource design. Data were collected at two time points with a 4-week interval. At Time 1, followers completed a questionnaire assessing their leaders’ humble leadership behaviors and their own altruistic value. All measurement instruments were administered in Chinese following the translation and back-translation procedure (cf. Brislin, 1986). Importantly, leaders were not informed that their leadership was being evaluated by followers at this stage to minimize potential social desirability or pressure-related biases. To ensure this, the survey materials explicitly emphasized confidentiality, and all responses were collected anonymously. At Time 2, followers rated their emotional experiences (i.e., elevation), and their direct leaders provided assessments of followers’ voice behavior. Leaders received only the section of the questionnaire relevant to follower behavior and did not have access to followers’ earlier responses.

At time point 1, we distributed 321 follower questionnaires and 62 leader questionnaires. A total of 313 valid follower questionnaires and 61 leader questionnaires were received (response rates of 97.51% and 98.39%, respectively). At time point 2, we received the same number of valid leader and follower questionnaires as for time point 1. Of the 313 leader-follower dyad questionnaires, 53.50% of the follower samples were male respondents, with an average age of 36.91 years and an average organizational tenure of 7.50 years. Among the leaders, 57.20% were male, the average age was 42.23 years, and the average organizational tenure was 13.35 years. Each leader rated between 1 and 13 followers, with an average of 5.13 followers per leader (SD = 2.88).

### 3.2 Measurements

Humble leadership was measured using a nine-item scale developed by Owens et al. (2013). Followers were considered to be more suitable evaluators for this variable (Rego et al., 2017). Items were rated on a six-point Likert scale (1 = strongly disagree, 6 = strongly agree). A sample question is: “My leader admits it when they do not know how to do something.”

Follower elevation was assessed using a nine-item scale from Algoe and Haidt (2009). We adopted this scale to capture followers’ recurring elevation experiences arising from leader–follower interactions using a six-point Likert scale (1 = not at all, 6 = very much). Followers were asked to indicate how frequently they experienced the following feelings, sensations, and desires when working with their leaders. A sample item is: “When you are working with your leader, how many times did you feel a sense of goodness or generosity.”

Follower altruistic *value* was assessed using the four-item altruistic value orientation scale adapted from De Groot and Steg (2008), originally based on Schwartz (1992). Participants rated the importance of specific values as guiding principles in their lives on a 9-point scale ranging from −1 (opposed to my values), 0 (not important) to 7 (extremely important). Sample items include “Equality (equal opportunity for all)” and “Social justice (correcting injustice, care for the weak).”

Follower voice was measured by leaders’ ratings of their followers’ voice behavior on a six-item voice scale from Van Dyne and LePine (1998). Responses were scored on a six-point Likert scale ranging from 1 (strongly disagree) to 6 (strongly agree). An example is: “This person develops and makes recommendations to me concerning issues that affect our organization.”

The reliability and validity of all constructs were assessed through Cronbach’s alpha, composite reliability (CR), average variance extracted (AVE), and standardized factor loading ranges. As shown in Table 2, all constructs demonstrated satisfactory reliability and convergent validity. Specifically, Cronbach’s alpha values ranged from 0.88 to 0.98, CR values ranged from 0.83 to 0.92, and all AVE values exceeded the recommended threshold of 0.50 (Fornell and Larcker, 1981). The standardized factor loading ranges are also reported in Table 2.

**Table 2:** Measurement properties of study constructs.

Construct	Cronbach’s *α*	CR	AVE	Standardized factor loading range
Humble leadership	0.98	0.91	0.64	0.679–0.879
Follower elevation	0.94	0.92	0.69	0.713–0.904
Altruistic value	0.98	0.83	0.63	0.789–0.795
Voice behavior	0.88	0.85	0.58	0.674–0.847

We included follower gender, age, education level, organizational tenure, and time under the current leader as control variables. Previous research suggests that demographic characteristics are related to followers’ voice behaviors (Detert and Burris, 2007). Follower organizational tenure was controlled because followers with longer tenure often have greater familiarity with organizational norms and networks, which can influence their comfort in raising potentially sensitive issues (e.g., Liu et al., 2022). The period of time for which a follower had worked with their current leader was also included, as extended leader–follower interactions may strengthen mutual understanding and trust, thereby affecting the follower’s likelihood of voicing (e.g., Tangirala and Ramanujam, 2008). Although it is impossible to obtain completely bias-free measurements, prior research has shown that followers’ ratings of leadership behaviors and voice behaviors are robust against various perceptual biases. For example, Brown et al. (2005) showed that employees’ perceptions of ethical leadership were not significantly affected by demographic factors, social desirability, or cynicism. Furthermore, substantial within-group agreement has been found in leadership ratings, suggesting these evaluations reflect a shared perception rather than individual biases. Therefore, perception-based assessments have been widely employed in leadership and employee behavior research (Liden et al., 2014; Mayer et al., 2012; Liu et al., 2012). Building on this evidence, our study relied on followers’ self-reports for humble leadership, follower elevation and follower altruistic value, and leaders’ ratings for follower voice behavior to mitigate common method bias and enhance the validity of findings.

### 3.3 Data analysis

Although this study employed a two-wave, multisource design, common method bias cannot be completely eliminated. Therefore, we adopted several procedural and statistical remedies to reduce its potential influence. First, following prior recommendations (Podsakoff et al., 2003; Podsakoff et al., 2012), we employed a two-wave survey design separated by a one-month interval. Specifically, humble leadership and altruistic value were assessed at Time 1, whereas follower elevation and leader-rated voice behavior were assessed at Time 2. Temporal separation between predictor and criterion variables can reduce consistency motifs and demand characteristics associated with common method bias (Podsakoff et al., 2003). Simultaneously, Ostroff et al. (2002) suggested that introducing a one-month time lag between measurements can reduce potential common method variance. Second, following the procedural recommendations of Podsakoff et al. (2003), participants were assured that their responses were anonymous and confidential, and they were informed that there were no right or wrong answers. In addition, questionnaire items were not arranged according to the hypothesized relationships among variables, thereby reducing respondents’ tendency to infer the research framework and provide consistent responses across measures (Podsakoff et al., 2003). Third, we conducted Harman’s single-factor test using confirmatory factor analysis (CFA) to assess potential common method bias (Malhotra et al., 2006; Podsakoff et al., 2003). The results showed that the hypothesized measurement model demonstrated substantially better fit than the one-factor model (see Table 3). These results suggest that common method bias was unlikely to substantially influence the interpretation of the findings (Podsakoff and Organ, 1986). Overall, these procedural and statistical remedies suggest that although common method bias cannot be completely ruled out, its potential influence on the interpretation of the findings is unlikely to be substantial.

**Table 3:** Confirmatory factor analysis of variables.

Model	Factors	χ^2^	df	χ^2^/df	CFI	TLI	RMSEA	SRMR	Δχ^2^	Δdf
1	4-factor model: HL; FE; FAV; FV	207.70	129	1.61	0.97	0.97	0.05	0.04		
2	3-factor model: HL + FAV; FE; FV	537.48	132	4.07	0.87	0.85	0.10	0.09	329.78^***^	3
3	2-factor model: HL + FAV; FE + FV	986.56	134	7.36	0.72	0.68	0.15	0.13	778.86^***^	5
4	1-factor model: HL + FE + FAV + FV	2006.86	135	14.87	0.38	0.30	0.22	0.21	1799.16^***^	6

HL, Humble leadership; FE, Follower elevation; FAV, Follower altruistic value; FV, Follower voice; CFI, comparative fit index; TLI, Tucker–Lewis index; RMSEA, root mean square error of approximation. ***p < 0.001.

Before hypothesis testing, we examined potential issues related to missing data, non-normality, and outliers. Missing data were handled using full information maximum likelihood (FIML) estimation. The distributions of the study variables were examined, and no substantial concerns regarding non-normality were identified. No cases with extreme values requiring exclusion were identified during data screening. Given the nested structure of the data and potential non-normality, all CFA and structural models were estimated in Mplus 8.0 using the robust maximum likelihood estimator (MLR), with leader ID specified as the clustering variable using the TYPE = COMPLEX approach.

The data analysis proceeded in four steps using Mplus 8.0 with a complex survey data approach to account for the nested leader-follower structure. Because multiple followers were nested within the same leaders, we first calculated intraclass correlations [ICC (1)] and design effects to assess the degree of non-independence among observations. The ICC (1) values were 0.000 for humble leadership, 0.126 for follower elevation, 0.099 for altruistic value, and 0.336 for voice behavior, with corresponding design effects of 1.000, 1.520, 1.411 and 2.388, respectively. To account for this nested structure, leader ID was specified as the clustering variable in Mplus 8.0 using the TYPE = COMPLEX approach with the robust maximum likelihood estimator (MLR). The data analysis proceeded in four steps. First, we assessed the composite reliability (CR), average variance extracted (AVE), and convergent validity of all latent constructs. Second, based on Anderson and Gerbing’s (1988) approach, confirmatory factor analysis (CFA) was conducted using a robust maximum likelihood method estimator to examine the distinctiveness of the assessed variables. Third, the direct effect of humble leadership on follower voice behavior (H1) and the mediating effect of elevation (H2) were tested. We used the Monte Carlo simulation method (20,000 resamples) to test mediation effect significance and constructed 95% confidence intervals using a software R-based simulator (Preacher et al., 2010). This method can accurately reflect the asymmetric nature of the sampling distribution of indirect effects. Finally, we conducted a moderated mediation analysis using a multi-group SEM approach to examine the moderating effect of altruistic value (H3) and the conditional indirect effect (H4). Specifically, follower altruistic value was operationalized as a grouping variable, and conditional indirect effects were compared between followers with low and high altruistic value.

## 4 Results

### 4.1 Confirmatory factor analysis

We conducted confirmatory factor analyses (CFAs) to examine the distinctiveness of the study constructs. The hypothesized four-factor model consisted of four separate factors: humble leadership, follower elevation, follower altruistic value, and follower voice behavior. As shown in Table 3, the four-factor model demonstrated good fit to the data (χ^2^/df = 1.61, CFI = 0.97, TLI = 0.97, RMSEA = 0.05, SRMR = 0.04) and significantly outperformed alternative models. Table 4 presents the means, standard deviations, correlations, and Cronbach’s *α* values of the variables examined in this study. In addition, correlations among latent variables estimated from the CFA model are reported in Table 5.

**Table 4:** Means, standard deviations and correlations.

Variables	Mean	SD	1	2	3	4	5	6	7	8	9
1. Follower gender^a^	0.54	0.500	–								
2. Follower age	36.91	9.309	0.071	–							
3. Follower educational level^b^	0.73	0.479	−0.027	−0.109	–						
4. Follower organizational tenure	7.503	6.688	−0.063	0.441^**^	0.162^**^	–					
5. Time under leader	4.937	4.798	−0.005	0.368^**^	0.091	0.607^**^	–				
6. Humble leadership	5.072	0.888	0.113^*^	−0.010	−0.067	−0.156^**^	−0.030	(0.98)			
7. Follower elevation	3.522	1.025	−0.039	−0.115^*^	0.051	−0.036	0.017	0.076	(0.94)		
8. Follower altruistic value	4.973	1.309	−0.042	−0.117^*^	0.052	−0.039	0.001	0.078	0.237^**^	(0.98)	
9. Follower voice	5.060	0.746	−0.065	0.017	−0.153^**^	−0.079	−0.067	0.146^**^	0.145^*^	0.090	(0.88)

*p < 0.05; **p < 0.01, two-sided test.

a 0 = female, 1 = male.

b High school or below = 0, junior college or bachelor’s degree = 1, master degree = 2.

α reliabilities are in brackets.

**Table 5:** Latent variable correlations.

Construct	1	2	3	4
1. Humble leadership	—			
2. Follower elevation	0.158	—		
3. Follower altruistic value	0.183	0.235	—	
4. Follower voice behavior	0.146	0.254	0.132	—

### 4.2 Hypothesis testing

The results of main effect (H1) testing are summarized in Table 6. Humble leadership was significantly positively correlated with follower voice behavior because the 95% CI did not contain zero (*γ* = 0.11, SE **=** 0.05, *p* < 0.05, 95% CI [0.008, 0.214] excludes zero) (Table 6). Hence, H1 was supported.

**Table 6:** Results of mediating and moderated–mediation effect.

Variables	Dependent variable					
	Follower elevation		Follower elevation		Follower voice	
	γ	*p*	γ	*p*	γ	*p*
Independent variable						
Follower gender	−0.200^*^	0.032	−0.201^*^	0.029	0.006	0.935
Follower age	−0.016^**^	0.002	−0.016^**^	0.002	−0.000	0.932
Follower educational level	0.010	0.911	−0.009	0.921	−0.124	0.056
Follower organizational tenure	0.005	0.613	0.002	0.846	−0.002	0.769
Time under leader	0.009	0.275	0.010	0.212	−0.003	0.636
Humble leadership (HL)	0.175^*^	0.016	0.172^*^	0.016	0.111^*^	0.035
Follower altruistic value (FAV)			−0.003	0.534		
HL × FAV			0.023^**^	0.002		
Follower elevation					0.124^**^	0.003
Indirect effects			Effect		LLCI	ULCI
			0.022		0.002	0.054
Conditional indirect effects			Effect		LLCI	ULCI
Follower altruistic value is high			0.065		0.018	0.158
Follower altruistic value is low			0.009		−0.023	0.058
Difference			0.056		0.003	0.148

95% confidence interval based on 20,000 Monte Carlo simulation samples.

Gender was coded: 0 = female, 1 = male.

Education was coded: High school or below = 0, junior college or bachelor’s degree = 1, master degree = 2.

*p < 0.05; **p < 0.01, two-sided test.

Regarding H2 (Table 6), the relationship between humble leadership and follower elevation was significantly positive (γ = 0.17, SE = 0.07, *p* < 0.05, 95% CI [0.033, 0.316] excludes zero), and a significant positive association was found between follower elevation and follower voice (γ = 0.12, SE = 0.04, *p* < 0.01, 95% CI [0.041, 0.206] excludes zero). We used the Monte Carlo method to construct confidence intervals by resampling. The indirect effect of humble leadership on follower voice through follower elevation was statistically significant (indirect effect = 0.022, Boot SE = 0.01, 95% CI [0.002, 0.054] excludes zero). These results supported H2.

**Figure 2:**
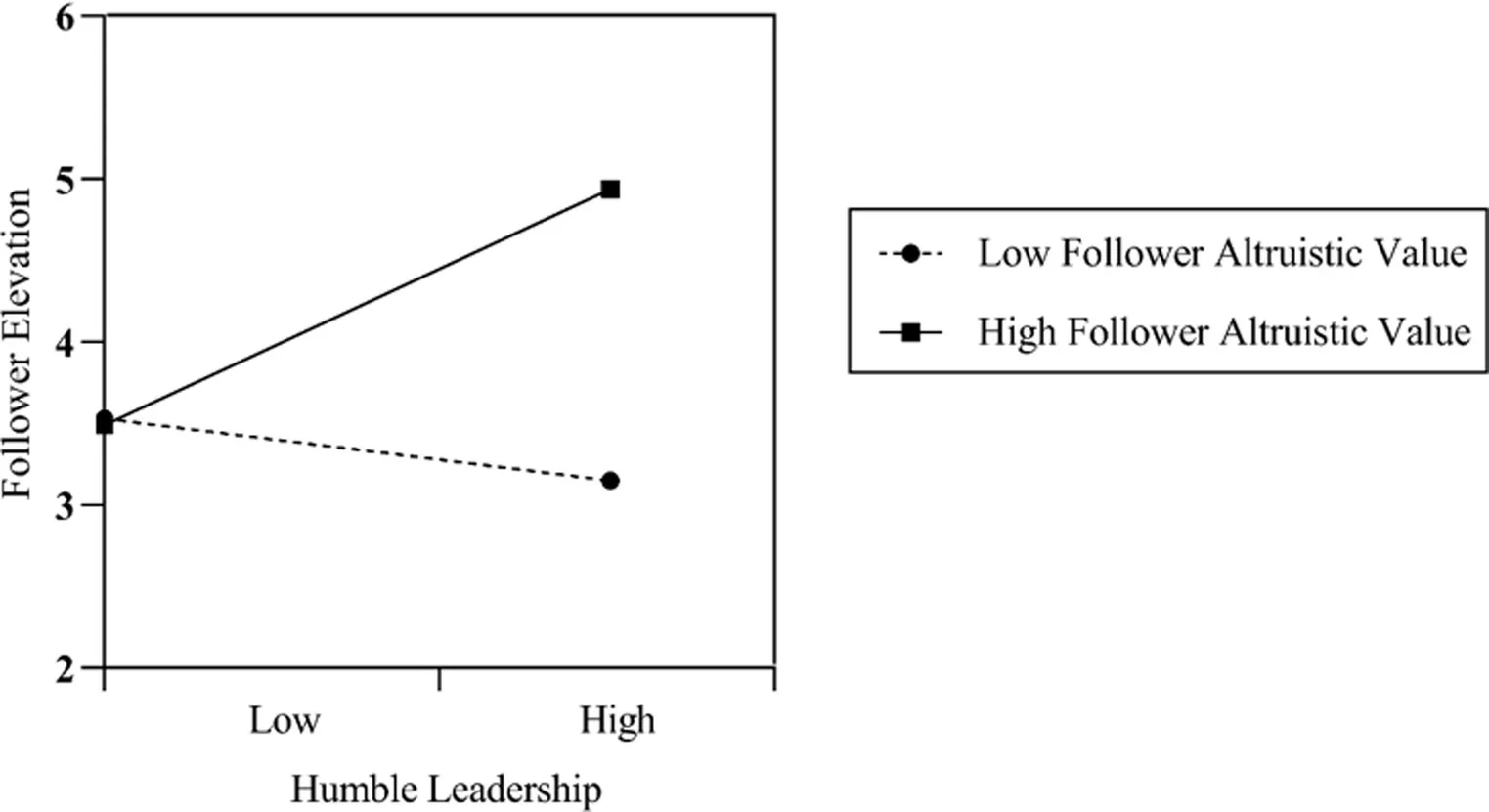
Moderating effect of follower altruistic value on the relationship between humble leadership and follower elevation.

Regarding H3, the interaction of humble leadership and follower altruistic value had a significant positive effect on follower elevation (γ = 0.02, SE = 0.01, *p* < 0.01, 95% CI [0.008, 0.037] excludes zero; Table 6). Regarding the types of interactions, the high and low moderator levels were defined as 1 SD above and below the mean, respectively (Figure 2). As expected, compared with followers with low altruistic value levels, humble leadership was more positively related to follower elevation in followers with high levels of altruistic value (effect = 0.241, SE = 0.07, *p* < 0.01, 95% CI [0.095, 0.387] excludes zero). Thus, H3 was supported.

Regarding H4, when follower altruistic value was high, the indirect effect was stronger (indirect effect = 0.065, 95% CI [0.018, 0.158] excludes zero) than when follower altruistic value was low (indirect effect = 0.009, 95% CI [−0.023, 0.058] includes zero). The difference between the two indirect effects was also positively significant (indirect effect difference = 0.056, 95% CI [0.003, 0.148] excludes zero). Thus, H4 was supported.

## 5 Discussion

This study responds directly to a call from Owens et al. (2013) that leaders must lead with “more humility and less hubris” (p. 1517) if they truly wish to hear more from their followers. Drawing on a multi-phase, multi-source design involving 313 leader–follower dyads in Chinese organizations—a context in which Confucian virtue ideals and high power-distance organizational norms may jointly shape the moral salience of humble leadership—our findings are consistent with a moral-emotional pathway linking humble leadership to follower voice. Specifically, our findings suggest that humble leadership is positively associated with follower elevation and that the indirect association between humble leadership and follower voice through elevation is consistent with the proposed mediation model. These findings identify elevation as a theoretically meaningful moral-emotional pathway that may help explain the association between humble leadership and follower voice.

Our work complements existing cognitive and relational explanations by introducing moral emotion as a complementary explanatory mechanism, reinforcing the importance of emotional dynamics in workplace behavior. Although previous studies have noted that negative emotions hinder voice (Ng and Feldman, 2012) and that positive emotions foster citizenship behavior (Koning and Van Kleef, 2015), few have identified specific moral emotions, like elevation, in the context of leadership influence. Our findings suggest that humble leadership may be associated with morally uplifting follower work experiences, which in turn may help explain follower voice behavior. Our results also found that follower altruistic value is a key boundary condition that shapes the effects of humble leadership. When followers possess strong altruistic values, they are more likely to feel elevated in response to humble leadership and to express their voice. This moderated mediation model supports the proposed conditional indirect association between humble leadership and follower voice through elevation, which appears stronger when followers report higher altruistic values.

### 5.1 Theoretical implications

This research provides several important theoretical contributions to the literature on humble leadership, follower moral emotions, and voice behavior, particularly within a non-Western context. First, our study advances humble leadership theory by identifying elevation as a complementary and relatively proximal self-transcendent moral-emotional pathway through which humble leadership is associated with follower voice behavior. While previous research has primarily explained the effects of humble leadership through cognitive and relational mechanisms, such as psychological safety, trust, empowerment, and identification (Kelemen et al., 2022), these perspectives mainly explain how humble leaders create supportive conditions that reduce barriers to follower participation. We do not view elevation as an alternative to these established explanations; rather, our model suggests that elevation operates alongside them by capturing the moral-emotional inspiration that may arise from followers’ perceptions of humble leaders’ moral exemplarity. Because follower voice requires employees to move beyond immediate self-interest and accept potential interpersonal risks for organizational improvement (Morrison, 2011; Tangirala et al., 2013), elevation provides a theoretically aligned pathway by activating moral aspiration, self-transcendence, and concern for collective welfare. Our findings extend this literature by suggesting that humble leaders influence followers not only by making voice behavior feel safer or more feasible, but also by inspiring followers through moral emotions. By identifying elevation as a self-transcendent moral emotion that connects followers’ perceptions of humble leaders’ moral exemplarity with their motivation to engage in voice behavior, this study extends humble leadership theory beyond instrumental and relational explanations. Specifically, our findings demonstrate how humble leaders may inspire followers to engage in voice through a moral-emotional process that matches the organization-oriented nature of voice behavior. Importantly, this contribution should not be interpreted as suggesting that emotional processes have been absent from humble leadership research, as prior studies have examined affective mechanisms such as gratitude and empathy; rather, our study refines this literature by focusing on elevation as a focal self-transcendent moral emotion that is theoretically aligned with the moral qualities of humble leadership and the prosocial nature of voice behavior.

Second, this study advances research on moral emotions in leadership by demonstrating the value of distinguishing specific moral emotions from broad affective valence. Previous research has largely focused on generalized positive affect (e.g., happiness) or negative affect (e.g., anger) when exploring emotion-behavior links (Koning and Van Kleef, 2015; Ng and Feldman, 2012). However, elevation represents a specific moral emotion because it is elicited by perceptions of virtue and prosocial excellence and involves a motivational orientation toward moral improvement and prosocial action (Algoe and Haidt, 2009). These distinctions further justify our theoretical positioning of elevation as a specific moral emotion, rather than a general positive affect. The motivational, cognitive, and behavioral consequences of elevation—particularly its emphasis on moral inspiration and prosociality—set it apart from other commonly studied emotions in leadership research, such as happiness or admiration (Algoe and Haidt, 2009; Siegel et al., 2014). By showing that followers’ responses to humble leaders involve not merely favorable affective reactions but moral inspiration, our study contributes to a more nuanced understanding of how specific moral emotions shape leadership outcomes beyond general emotional valence. This perspective complements emerging research on moral emotions in organizational contexts (Hericher et al., 2023; Kruse et al., 2014; LaBouff et al., 2012).

Third, our moderated mediation findings shed light on the often-overlooked role of follower values in shaping leadership effectiveness. While recent research has increasingly recognized the role of follower characteristics, such as individual-level power distance orientation, in moderating the impact of humble leadership (e.g., Qian et al., 2018), empirical attention regarding how specific follower values shape emotional and behavioral reactions to leadership remains limited. Our results suggest that follower altruistic value strengthens both emotional and behavioral pathways. In our case, followers’ altruistic values were more likely to be activated by humble leadership, showing stronger elevation and subsequent voice behavior. By highlighting the enabling role of follower altruistic value, we contribute to a more person-centered account of how humble leadership operates.

Fourth, by situating our study in the Chinese organizational context, we offer a culturally grounded theoretical interpretation of the humble leadership–elevation–voice pathway. We clarify that our findings based on Chinese organizations should not be interpreted as empirical evidence of cultural specificity in the absence of a comparative cultural design. Instead, we highlight how two cultural logics may coexist. On the one hand, high power-distance organizational norms may lead followers to expect leaders to assert authority and maintain hierarchical distance (Farh et al., 2007; Hu et al., 2018), making humble behaviors relatively unexpected in everyday leader–follower interactions. On the other hand, Confucian traditions value humility, moral self-cultivation, and the ideal of the morally exemplary person (Chan, 2015; Wang and Xiang, 2025), providing followers with a cultural schema for interpreting leader humility as moral excellence. Thus, humble leadership may be both unexpected in routine hierarchical practice and morally valued at the level of cultural ideals. This distinction helps theorize why humble leadership may be theoretically salient as a moral event in Chinese organizations. By explicating these culturally embedded processes, we respond to calls to integrate cultural values into leadership theorizing (Luo et al., 2022).

### 5.2 Practical implications

This study offers actionable guidance for promoting voice behavior in high-power distance contexts by leveraging moral emotions. First, organizations should prioritize humility in leadership selection. Beyond technical competence, firms can use multi-source feedback to identify candidates who demonstrate openness and appreciation for others. Our findings confirm that humble leaders act as moral exemplars whose behaviors are associated with followers’ elevation experiences and willingness to speak up.

Second, leadership development programs may encourage leaders to display humble behaviors that foster positive moral-emotional experiences among followers. Specifically, acknowledging personal limitations, appreciating followers’ contributions, and demonstrating openness to learning are important manifestations of humble leadership (Owens and Hekman, 2012; Owens et al., 2013). Such behaviors may help followers perceive leaders as moral exemplars and become more willing to contribute constructive ideas.

Finally, organizations may consider providing social recognition for employees who engage in constructive voice behavior. Because voice behavior often involves interpersonal risks (Morrison, 2011), acknowledging employees’ constructive intentions and contributions may help create a more supportive environment for suggesting.

### 5.3 Limitations and future directions

First, although this study employed a two-wave, multi-source design, the simultaneous measurement of follower elevation and voice behavior at Time 2 limits our ability to establish the temporal ordering between elevation and voice. Therefore, the findings should be interpreted as consistent with the proposed mediation model rather than as definitive evidence that elevation causes voice behavior. Future research could adopt three-wave designs or experience-sampling approaches to capture the temporal dynamics of this emotional process more precisely. Relatedly, although our elevation measure captures followers’ recurring elevation experiences arising from leader–follower interactions, it does not fully capture momentary elevation episodes elicited by specific leadership events. Consistent with affective events theory (AET; Weiss and Cropanzano, 1996), future research could employ event-contingent or experience-sampling designs to examine how specific humble leader behaviors trigger immediate elevation responses and how these emotional episodes subsequently influence follower behavior. Second, although the Chinese organizational context provides a theoretically meaningful setting for examining humble leadership and elevation, prior research on humble leadership has primarily focused on defining the construct, examining its mechanisms, and documenting its consequences, while the contextual conditions under which humble leadership operates remain an important area for further investigation (Kelemen et al., 2022). However, our single-country design limits conclusions regarding cultural specificity and cross-cultural differences. Therefore, the cultural arguments developed in this study should be interpreted as contextual theorizing rather than empirical evidence that the humble leadership–elevation–voice pathway is stronger in China than in other cultural contexts. Future comparative research could examine whether Confucian virtue schemas, power-distance norms, or other cultural values shape this moral-emotional pathway. Third, we highlight elevation as a specific moral emotion. Given that individual behavior stems from complex affective profiles (Mindeguia et al., 2025), future scholars should disentangle “other-praising” emotions (elevation) from “benefit-based” emotions (gratitude). This would clarify how distinct leadership signals activate specific prosocial versus reciprocal behaviors. Fourth, our cross-industry design may obscure sector-specific dynamics. Future research should replicate this model in sectors with varying “moral salience” (e.g., healthcare vs. finance) to examine if industry norms moderate the moral-emotional influence of humility. Finally, we restricted our boundary condition to follower values. Future studies should investigate leader-follower value congruence, as shared moral schemas may be necessary for humble leadership to function as a transformational moral event.

## 6 Conclusion

This study advances understanding of why humble leadership encourages follower voice by identifying elevation as a central moral-emotional mechanism and follower altruistic value as an important boundary condition. Drawing on Affective Events Theory, our findings show that humble leadership functions as a morally salient event that elicits follower elevation, which in turn motivates followers to engage in voice behavior. Moreover, this indirect effect is stronger among followers who hold higher altruistic values, suggesting that the influence of humble leadership depends not only on leaders’ behaviors, but also on followers’ value orientations. By moving beyond cognitive and exchange-based explanations, this study highlights moral emotion as a complementary pathway through which humble leadership fosters a risky yet prosocial form of employee behavior. Situated in the Chinese organizational context, the findings further suggest that humble leadership may carry heightened moral meaning in hierarchical settings shaped by Confucian values. Practically, organizations may cultivate voice more effectively by selecting and developing leaders who display humility in visible ways, and by reinforcing the prosocial values and moral motivation that encourage followers to speak up. Rather than relying primarily on transactional incentives, organizations may benefit from treating voice as a morally meaningful contribution to collective improvement.

## Data Availability

The raw data supporting the conclusions of this article will be made available by the author, without undue reservation.
